# Lessons learned from open laryngotracheal airway resection and primary anastomosis in high risk patients

**DOI:** 10.1371/journal.pone.0238426

**Published:** 2020-09-21

**Authors:** Karuna Dewan, Gerald S. Berke, Dinesh K. Chhetri

**Affiliations:** 1 Department of Head and Neck Surgery, Stanford University, Stanford, CA, United States of America; 2 Department of Head and Neck Surgery, David Geffen School of Medicine at UCLA, Los Angeles, CA, United States of America; Sapienza University of Rome, ITALY

## Abstract

**Objective:**

Laryngotracheal stenosis is one of the most difficult conditions treated by the Otolaryngologist. Open resection of stenosis with primary airway anastomosis is the definitive treatment for this condition. However, some patients are considered high risk candidates for open airway surgery and management and outcomes in this group have not been reported. The purpose of this investigation is to identify a series of high risk patients who underwent open laryngotracheal surgery and detail the lessons learned in regards to their post-operative course and outcomes.

**Methods:**

A retrospective cohort study of all patients that underwent airway resection and primary anastomosis over a fifteen-year period was performed. High-risk patients, those with medical comorbidities that impair wound healing, were identified. Post-operative course, management of complications, and ultimate airway outcomes were noted.

**Results:**

Seven patients fitting the high-risk category were identified. Comorbidities were poorly controlled insulin dependent diabetes mellitus (N = 4), poorly controlled hypertension (N = 4), end stage renal disease requiring hemodialysis (N = 3), chronic obstructive pulmonary disease (N = 1), and history of radiation therapy (N = 1). Each patient suffered postoperative complications of varying degrees including postoperative infection (N = 1), formation of granulation tissue at the anastomotic site (N = 3), and postoperative hematoma (N = 1). Management included treatment of infection and complications. Anastomotic dehiscence was managed with tracheostomy and T-tubes.

**Conclusions:**

High-risk medical comorbidities may not be absolute contraindications for open laryngotracheal resection of airway stenosis. However, this experience emphasizes the importance of preoperative medical optimization and comprehensive postoperative care.

## Introduction

Laryngotracheal stenosis is characterized by segmental airway stenosis involving the subglottic larynx and trachea. It is often the result of direct injury to the tracheal cartilage or inflammatory granulation tissue causing airway narrowing, most commonly secondary to luminal trauma such as prolonged intubation [1]. It is also one the most difficult conditions treated by the otolaryngologist. Factors that must be considered in selecting the treatment modality for laryngotracheal stenosis include the length, location, and severity of stenosis. Short-segment stenosis that is less than or equal to Cotton-Myer grade 3 narrowing may best be managed endoscopically with laser ablation, dilation, steroid injection and/or mitomycin C application [2, 3]. Unfortunately, a major disadvantage of endoscopic management is a high rate of restenosis and need for repeated surgery.

Common open surgical treatment options for airway stenosis include laryngotracheoplasty (LTP), tracheal resection (TR), and cricotracheal resection (CTR). Unlike endoscopic resection, open airway resection has the advantage that only one definitive surgery may be needed to treat the stenosis. However, it carries an increased risk of significant surgical complications and morbidity such as anastomotic dehiscence. Due to the inherent increased risk of open airway surgery, some patients are considered high risk candidates. High risk candidates are those who may possess any number of identified potential risk factors for poor wound healing. Wright et al. reported that reoperation, lengthy resection, diabetes, age seventeen years or younger, and preoperative tracheostomy status were predictive of anastomotic complications [4]. Diabetes mellitus is the most common metabolic disease associated with impaired wound healing, although it is not clear to what extent the impaired healing is due to the direct effects of insulin deficiency or its sequelae. Although Wright et al. address some of the predictors of increased complications in open airway surgery the procedures described are primarily thoracic airway procedures as well as more extensive procedures than those typically performed by otolaryngologists via the cervical approach. Within the Otolaryngology literature there are no reviews or discussions of those patient risk factors which are associated with increased complication rates following open airway surgery and therefore some guidance on the optimal management of airway stenosis in high risk patients is needed.

The purpose of this study is to identify a series of high risk patients who underwent open laryngotracheal surgery and detail their post-operative course and outcomes. We detail the lessons learned and management of perioperative complications. By reviewing these risk factors and discussing their perioperative management, we hope to further highlight the challenges and ways to manage the airway in these patients who are often deemed poor surgical candidates and often not offered open surgery.

## Methods & materials

This study protocol was approved by the institutional review board. From a single-institution Otolaryngology patient database, a patient list was gathered by identifying specific International Classification of Diseases (ICD-9) diagnosis codes (519.19, 748.3) over a fifteen-year period. A retrospective review was performed for all identified patients that underwent airway resection and anastomosis for laryngotracheal stenosis. Within this group the high risk patients were identified. Patients were deemed to be high risk if they had the following medical co-morbidities: insulin dependent diabetes mellitus, chronic hypertension, end stage renal disease requiring dialysis, chronic obstructive pulmonary disease, and history of radiation therapy to the neck. These risk factors were chosen as they are all factors known to impact wound healing and/or scar formation. The current study includes patients undergoing surgery between January 2000 and January 2016. Demographics, clinic notes, operative reports, hospital records and follow-up data were recorded. Patients were included in the study if they were at least 17 years old and if they had airway resection of at least 6 rings length with primary anastomosis. Post-operative complications and ultimate airway outcomes were reviewed.

Demographic and clinical data recorded included age at the time of presentation, sex, etiology of stenosis, prior intubation, past medical history and smoking history. Prolonged intubation was defined as any intubation greater than 24 hours, and symptoms of stenosis were temporally associated. Stenosis was determined to be idiopathic if there was no other underlying cause of pathologic abnormalities, including trauma, diagnosis of autoimmune disease, or history of intubation. Stenosis grade was based upon the Meyer-Cotton grading system (grade I< 50% stenosis; grade II 51%-70% stenosis; grade III, 71%-99% stenosis; and grade IV complete stenosis with no detectable lumen) [5]. Both grade and length of stenosis were determined based upon descriptions from intraoperative tracheobronchoscopic findings at the time of airway resection.

Cricotracheal resection was performed similarly to previous reports [6–8] in a single-stage technique. Tension-relieving procedures were employed as needed, which included laryngeal release, cervical tracheal mobilization, or mediastinal tracheal mobilization to the carina. Post-operatively neck hyperextension was prevented by slightly flexing the neck and placing a suture securing the chin to the chest (“Grillo” stitch) at the conclusion of the case. This stitch was kept for 5 to 7 days depending on the degree of tension at the primary anastomosis site. For two of the patients, tracheostomy was performed below the level of the anastomosis at the conclusion of CTR, and decannulation occurred after hospital discharge.

Ethical Considerations: This study is a retrospective review. The risks to the patients are minimal as their data has been deidentified. Charts of all 21 patients undergoing laryngotracheal resection with primary anastomosis during a 15-year period were reviewed. Therefore, selection bias within the cohort is not an issue. However, this study was performed at a tertiary referral center. These cases are likely more complex than those seen by a general otolaryngologist.

## Results

Twenty-one patients that underwent laryngotracheal resection with primary anastomosis were identified from the fifteen years study period. Seven subjects fit the high risk criteria. The remaining fourteen patients did not have significant pre-existing medical conditions and did not suffer any major post-operative complications. The high-risk cohort was 86% male. The mean age at the time of surgery was 58 years (range 37–72). The mean duration of follow-up from the time of surgery was 2.6 years (range 0.2–5.2). The etiology of stenosis is detailed in Table 1. Causes of laryngotracheal stenosis included prolonged intubation (71%) or a history of prior tracheostomy (29%). 6 of the 7 (86%) had failed several attempts at endoscopic treatment. Grade of stenosis at the time of open surgery was grade 3 in 71% and grade 4 in the rest. At the time of resection 3 of 7 (43%) had a tracheostomy. At last follow up 5 of 7 (71%) were successfully decannulated; the remaining two were tracheostomy or T-tube dependent. The average length of resection was 3.4 cm (range, 2.5–4). 71% of resections included the cricoid.

**Table 1:** Description of patients included in this study.

Patient No.	Age (Years)	Comorbidities	Previous Treatment	Stenosis Grade	Resection Length (cm)	Complications
**1**	37	ESRD	Endoscopic laser, dilation, steroid and Mitomycin-C	3	3	MRSA infection
**2**	61	ESRD, DM, MI	None	4	4	Minimal re-stenosis
**3**	47	ESRD, HTN	Endoscopic laser, dilation, steroid and Mitomycin-C	3	4	Post-op hematoma
**4**	69	XRT, DM, CAD	Endoscopic laser, dilation, steroid and Mitomycin-C	3	3	Anastomosis breakdown- required tracheostomy
**5**	65	COPD	Endoscopic Dilation	3	3	Aspiration, Permanent tracheostomy
**6**	56	DM	Endoscopic laser, Tracheostomy,	3	4	Granulation tissue
**7**	72	DM, PVD, MI	Tracheostomy	4	4	Permanent T-tube needed

IDDM: Insulin dependent diabetes mellitus, ESRD: End stage renal disease, MI: Myocardial Infarction, HTN: hypertension, CAD: coronary artery disease, COPD: Chronic obstructive pulmonary disease, PVD: Peripheral vascular disease.

Post-operative complications included infection, formation of granulation tissue at the anastomotic site and hematoma (Table 1). Three patients required postoperative endoscopic dilation. There were no mortalities in this series. Four illustrative cases are presented below:

### Case 1

**Fig 1:**
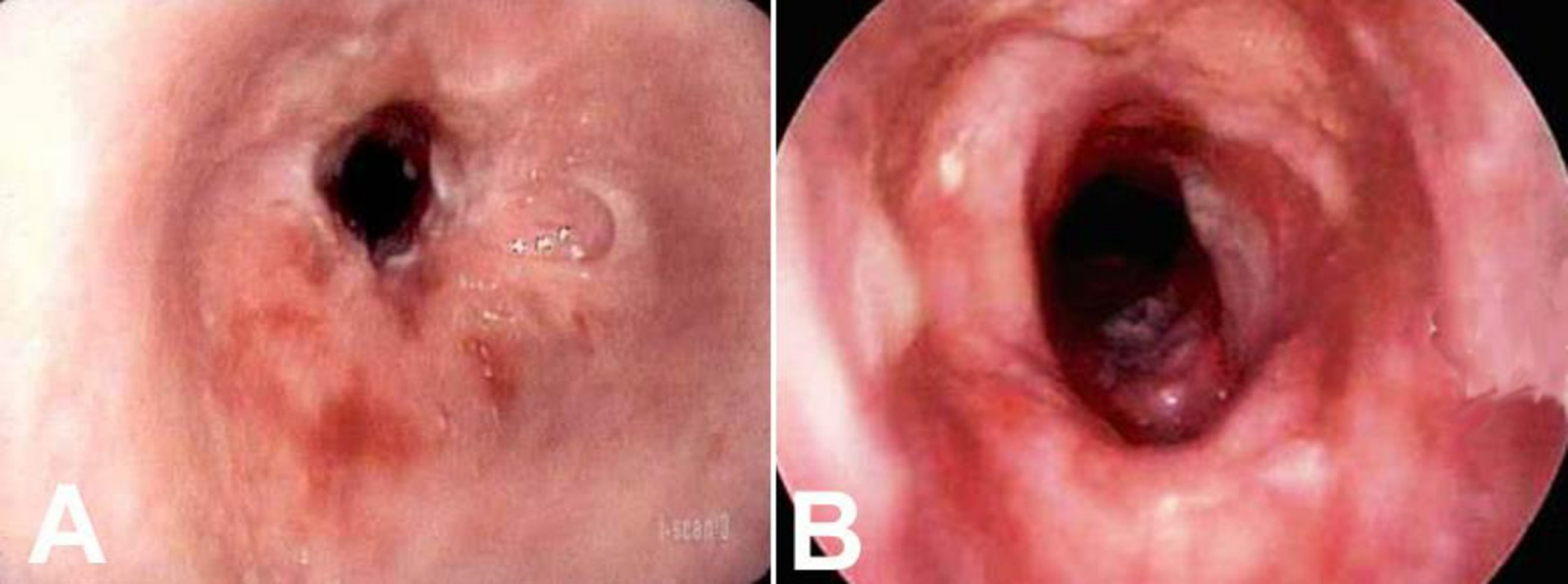
A—Preoperative endoscopy of case 1 demonstrates circumferential Grade 3 stenosis 2 cm long involving the cricoid. B—Postoperative endoscopy after T-tube removal one year after initial resection.

Patient No. 1 was a 35 year old male who was cleaning inside a chemical tank that exploded. He required prolonged ventilation and was extubated but he developed end stage kidney disease requiring hemodialysis three times per week. He also suffered from myocardial infarction and had poorly controlled insulin dependent diabetes mellitus. He presented to outpatient clinic six months later with stridor and was noted to have 2 cm long and thick tracheal stenosis that did not respond to two endoscopic treatments (Fig 1A). Thus, he subsequently underwent a technically unremarkable tracheal resection involving 3 tracheal rings, with primary anastomosis. Post-operative medications included intravenous clindamycin and dexamethasone, which was converted to Medrol dosepack on postoperative day (POD) #2. On POD#3, he developed neck erythema that rapidly progressed over 12 hours to stridor, fevers, and tachycardia. He underwent urgent neck exploration and was found to have hematoma and granulation tissue. The anastomosis appeared intact, withstood a leak test, and was covered with the thyroid gland and therefore was left untouched. Cultures from the wound as well as nares and the recently placed Hickman site grew methicillin resistant staphylococcus aureus (MRSA) and antibiotics were appropriately changed by infectious disease specialists. At the conclusion of the procedure the patient was left intubated for 3 days. Upon extubation he did well for less than a day and thus a repeat washout of the neck and a tracheostomy was performed. The tracheostomy was placed through the anastomosis site and a stomaplasty was performed around this site to secure the stoma. The tracheostomy was converted to a T-tube nine days later. After discharge the patient was treated with hyperbaric oxygen to improve healing and he underwent continued hemodialysis. The T-tube was removed and tracheocutaneous fistula closed six-months later.

### Case 2

**Fig 2:**
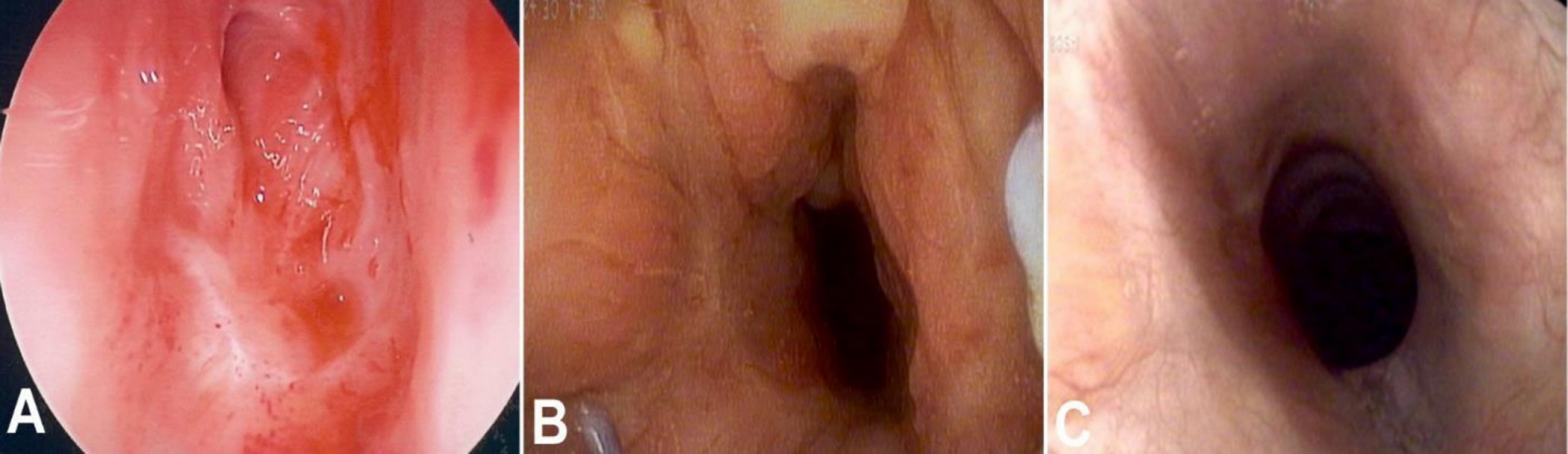
A—Preoperative endoscopy of case 2 demonstrates a Grade 4 stenosis involving the cricoid and 5 tracheal rings. B- Postoperative endoscopy with edema 6 weeks after resection. C- Postoperative endoscopy six months after surgery with resolution of edema.

Patient No. 2 was a 61 year old male with a history of diabetic ketoacidosis leading to prolonged intubation. His past medical history included poorly controlled insulin dependent diabetes mellitus, end stage renal disease requiring dialysis and history of myocardial infarction. He presented with a tracheostomy and was noted on endoscopic exam to have a grade IV stenosis (Fig 2A). He underwent cricotracheal resection involving 4 tracheal rings and the cricoid with primary anastomosis. On POD#1 he required reintubation for respiratory failure due to right lower lobe pneumonia. On POD#4 he was extubated and then reintubated for continued respiratory distress and inability to clear secretions. His blood sugars were poorly controlled throughout the hospital stay and averaged over 300 for the first 3 days after surgery. On POD#7 an endoscopic exam performed in the operating room showed intact anastomotic site with edema of the vocal folds and trachea but a reasonable airway and thus a tracheostomy was not performed. The patient was definitively extubated on POD#9 and discharged on POD#15. Post-operatively the edema at the anastomotic site decreased slowly (Fig 2B). At his last visit one year after airway resection his airway edema finally appeared resolved (Fig 2C).

### Case 3

**Fig 3:**
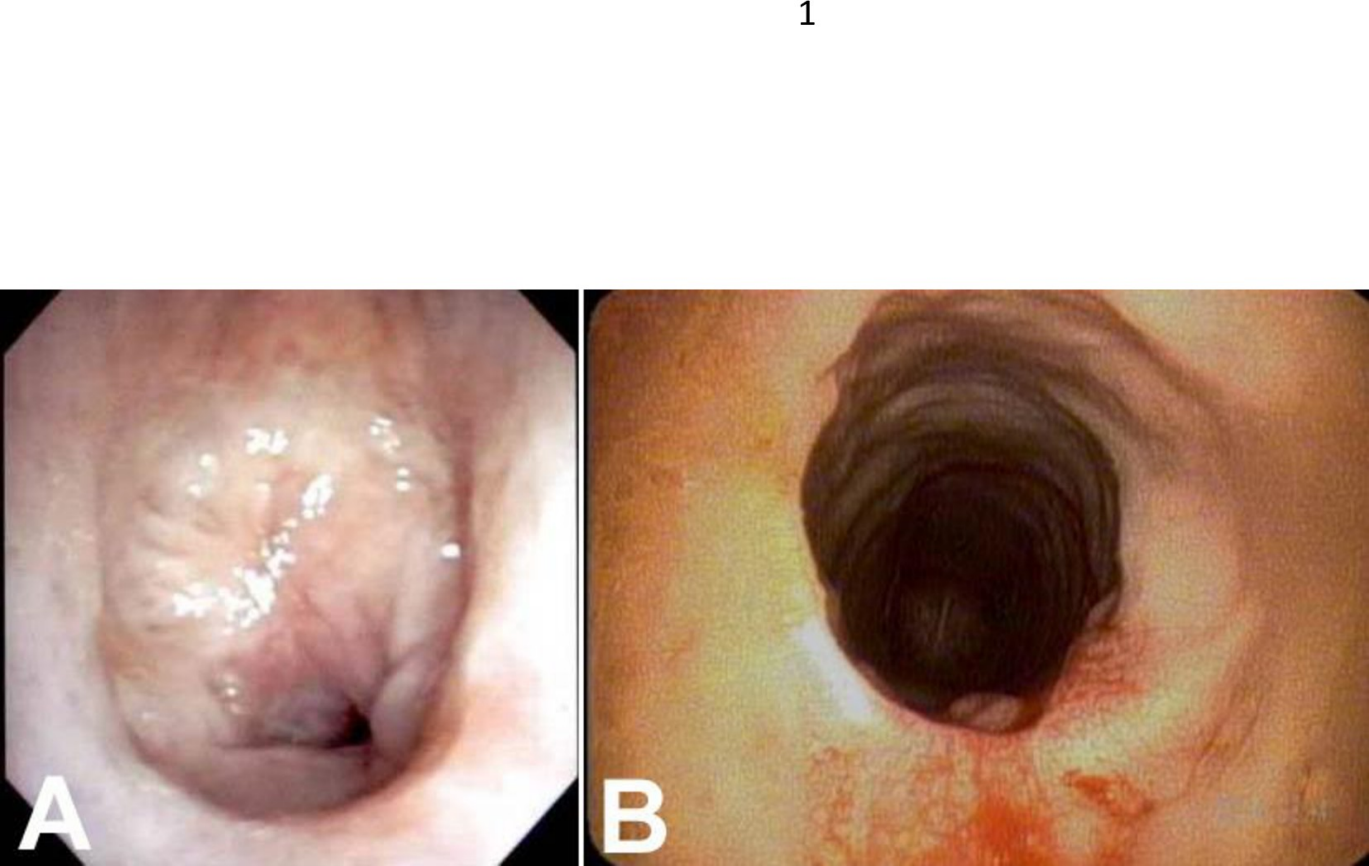
A—Preoperative endoscopy of case 3 demonstrates a circumferential grade 3 subglottic stenosis involving 3 tracheal rings. B—Postoperative endoscopy six weeks after resection shows a resolution of stenosis.

Patient No. 3 was a 46 year old male with uncontrolled hypertension, congestive heart failure, and end stage renal disease requiring hemodialysis. He developed grade 3 subglottic and tracheal stenosis after intubation for fluid overload and respiratory distress (Fig 3A). Endoscopic treatments were inadequate and he remained symptomatic. The patient underwent a 3.5cm airway resection with primary anastomosis. Poor tissue quality with tracheomalacia was noted. Postoperatively he suffered from uncontrolled hypertension requiring a Nicardipine drip. On POD#3 he developed sudden neck swelling with respiratory distress and required neck exploration for drainage of a hematoma. He subsequently did well and was discharged home on POD#11. He had persistent edema at the anastomotic site that resolved over the next six weeks (Fig 3B).

### Case 4

Patient No. 4 was a 69 year old male with a history of laryngeal cancer treated with radiation therapy several years prior to presentation. He required a tracheostomy at the time of his cancer diagnosis but was subsequently decannulated after therapy. His past medical was significant for coronary artery disease requiring stenting, hypertension and uncontrolled insulin dependent diabetes mellitus. He developed right angle torsion and subluxation of trachea at the site of his tracheostomy site that was not amenable to endoscopic resection. He underwent cervicothoracic tracheal resection involving the cricoid and 3 tracheal rings, with primary anastomosis. On POD#3 he developed an anastomotic leak. He was taken to the operating room with the intent to place a tracheostomy through the dehiscent trachea but the anastomosis was noted to abut the innominate artery. Therefore, the tracheal dehiscence was primarily repaired and covered with sternohyoid muscle advancement and he was left intubated with the cuff past the anastomosis. A repeat bronchoscopy on POD#6 revealed intact anastomotic site but he failed extubation trial due to development of supraglottic edema from prolonged intubation and inability to clear secretions and a tracheostomy was performed. The tracheostomy incision was made vertically and encompassed the airway anastomotic site and a stomaplasty was performed to protect the innominate artery and to support the tracheal anastomosis. He subsequently underwent one endoscopic treatment 4 months after airway resection and was decannulated successfully. The patient continues to do well with mild grade 1 stenosis 5.5 years later.

There were two patients who could not be decannulated (Table 1). Patient 5 had somewhat poorly controlled COPD and a history of tracheal stenting, a procedure that predisposes patients to re-stenosis at the level of the stent [9]. He was noted to aspirate after resection. Therefore, a tracheostomy was placed for pulmonary toilet, and the anastomosis eventually restenosed completely. He prioritized eating over speaking and no further intervention was performed. Patient 7 was a 72 years old male who developed airway stenosis after an emergent slash tracheostomy using a vertical incision. Advanced age is identified by several studies as a risk factor for poor surgical outcome after tracheal resection. At the time of resection, the condition of the tracheal wall was very poor and the cricoid plus 6 tracheal rings were resected. Postoperatively, he developed an infection resulting in anastomotic dehiscence. He was taken back to the operating room for debridement of the necrotic anterior tracheal wall and for tracheostomy placement. The tracheostomy was eventually replaced with a T-tube. After 5 years with the T-tube the patient was lost to follow up.

## Discussion

Laryngotracheal stenosis remains one of the most challenging conditions to treat in Otolaryngology. This study is first comprehensive case review of airway resection in a high risk group in the otolaryngology literature. The cases presented here demonstrate that patients considered “high risk” for airway resection due to the presence of certain comorbidities, may actually do well ultimately. However, post-operative challenges and complications are to be routinely expected and the control and management of these comorbid conditions during the immediate postoperative period is paramount to surgical success and good outcomes. While medical comorbidities including poorly controlled insulin dependent diabetes mellitus, poorly controlled hypertension, end-stage renal disease requiring dialysis, COPD/Asthma, and previous radiation therapy exposure impair healing, they may not have to be absolute contraindications to airway resection with primary anastomosis. Preoperative medical optimization and comprehensive postoperative care can lead to good outcomes in this population.

In a retrospective review Lano et al. identified many factors linked to difficulty in postoperative decannulation. Among those were a history of obstructive sleep apnea, recurrent pneumonia, and congestive heart failure. Several studies proposed advanced age as a predictor for surgical failure [10], defined as failure to decannulate within one year of surgical repair. Systemic hypertension is considered high risk due to its contribution to pathological scarring and poor wound healing mediated by inflammation and hypoxia. In this way it is an aggravating/risk factor for the development of hypertrophic scarring and recurrence of stenosis and is associated with pro-fibrotic functional changes in cells involved in healing including endothelial cells, pericytes/myofibroblasts, dermal fibroblasts and mast cells [11]. Radiation exposure to the larynx and trachea comprise a notable risk factor for surgical failure as irradiated tissues demonstrated impaired capillary regeneration and decreased rates of fibroblastic proliferation. Necrosis may develop after surgical manipulation of the trachea as capillary blood flow is compromised by hyalinization after radiation exposure [12–15]. Asthma and COPD are chronic inflammatory conditions that lead to remodeling of both small and large airways and reduce cartilage integrity, thus patients with these two comorbid conditions are high risk for complications in airway reconstruction [16, 17]. End stage renal disease or major renal impairment is widely believed to have negative implications for wound healing. Uremic toxins that are poorly excreted and accumulate in tissues negatively impact local mechanisms of wound healing [18]. Chronic kidney disease promotes the development of a chronic inflammatory state. This leads to poor wound healing secondary to slowed rates of neovascularization and cell proliferation. Murine research implies that impaired keratinization, delayed granulation and large epithelial gaps justify the perilous impact of chronic kidney disease on wound healing [19]. Human data, similar to that in animal studies indicates that patients with chronic kidney disease suffer increased rates of wound disruption as compared to those patients with a normal glomerular filtration rate [20].

Post-operative complications appear to mirror the underlying comorbidity. Patients with uncontrolled hypertension are at increased risk for postoperative hematoma and require adequate blood pressure control. COPD/asthma, insulin dependent diabetes mellitus and end stage renal disease are all conditions associated with increased systemic inflammation and poor wound healing. In these patients, one must be vigilant to recognize poor anastomotic healing, which may be exacerbated by infection, increased tension at the anastomosis, or history of prior radiation therapy. If anastomotic dehiscence does occur after tracheal resection, it is preferable in our practice to perform the tracheostomy through the anastomotic site. A stomaplasty is performed as well, securing the anterior neck skin circumferentially to the tracheal cartilage to support healing by reducing tension on the suture line. This strategy prevents restenosis at the anastomotic site if a tracheostomy is placed below the anastomosis. Subsequently, the tracheostomy can be replaced with a T-tube if healing remains poor or further airway stenting is necessary. The T-tube prevents the development of significant stenosis while allowing the patient to voice. This technique was successfully used in three patients.

Further lessons were gleaned regarding the issue of intubation after airway resection and primary anastomosis. In our practice, we prefer to extubate the patient after surgery because it is generally thought that presence of endotracheal tube may place undue pressure on the anastomosis. However, two patients in this cohort required postoperative intubation. Patient No. 2 required intubation for 1 week post-operatively and healed well at the anastomosis site. While patient No. 4 was kept intubated after repair of tracheal dehiscence and did not tolerate extubation, it was due to supraglottic edema predisposed by prior radiation therapy to the larynx and not narrowing or other complications at the anastomosis. Thus, tracheal intubation postoperatively appears not necessarily deleterious to healing or subsequent decannulation. We found that in this population with significant medical comorbidities resulting in impaired healing possible reintubation post-operatively should be anticipated.

Another important finding is that in this high-risk group, further endoscopic treatment after airway resection may be needed and allows for further improvement in airway caliber. Those who have delayed or abnormal healing at the anastomotic site will develop fibrotic healing and airway stenosis that is manageable endoscopically. There are several limitations to this study. This study is a retrospective review of cases with their associated limitations. In addition, the sample size is small. However, the clinical scenarios presented here have not been detailed before and the numbers are generally small because patients with significant comorbidities are generally not offered airway resections.

## Conclusion

Patients with significant comorbidities, those previously deemed to be poor candidates, can be offered airway resection with primary anastomosis. However, post-operative complications are to be expected and managed accordingly.
